# Seismic Damage Evaluation of Beam-Column Joints in Monolithic Precast Concrete Frame Structures

**DOI:** 10.3390/ma15176038

**Published:** 2022-09-01

**Authors:** Yan Cao, Zhao Yang

**Affiliations:** 1 School of Arts Design, Wuchang University of Technology, Wuhan 430223, China; 2 School of Urban Construction, Wuhan University of Science and Technology, Wuhan 430065, China

**Keywords:** seismic damage evaluation, beam-column joint, monolithic precast concrete frame structure, cyclic loading, damage index

## Abstract

Quantitative calculation and evaluation of seismic damage are very important for structural safety, performance-based structural analysis, and seismic reinforcement. However, the relevant research results for precast concrete structures are extremely limited. In this paper, the seismic damage evaluation of beam-column joints in monolithic precast concrete frames was studied through cyclic loading tests and damage index calculation. The seismic damage process, load-displacement relationship, stiffness degradation, and the influence of axial compression ratio were analyzed, then the damage indexes were calculated and analyzed, and the quantitative evaluation of joint damage was conducted last. The results show that the connection seams can significantly affect the mechanical properties of precast joints, easily causing damage concentration, resulting in a lower bearing capacity and faster stiffness degradation compared with a cast-in-situ joint. A larger axial compression ratio can bring higher bearing capacity for the precast joints, and the peak load can be increased by 42.9% when the axial compression ratio is increased from 0.2 to 0.4. In contrast, the stiffness degradation will be accelerated with the increase in the axial compression ratio. From yield load to peak load, the stiffness of the precast joint with the largest axial compression ratio decreases by 46.0%, while the joint with the smallest axial compression ratio is only 36.4%. The damage index model adopted in this paper can accurately reflect the damage characteristics of the precast joints. The presented damage states based on the damage index calculation can accurately reflect the joint’s damage characteristics according to different stages. The paper realizes the quantitative damage evaluation for this kind of joint and provides a theoretical basis and method for further studies.

## 1. Introduction

Structures are prone to damage under seismic action. Accurate evaluation of seismic damage can provide a reliable basis for structural identification and repair and has important research value. The damage process of the structure under an earthquake is essentially cumulative damage. Many scholars have studied the damage to concrete structures. According to the actual seismic damage results, the seismic damage forms are divided into the first-passage failure and the cumulative damage failure, and a single-parameter model and a two-parameter model are proposed to evaluate the damage degree of structural members. Among them, the single parameter model mainly adopts one parameter index, such as deformation [1,2], ductility [3,4], failure ratio [5], or energy dissipating capacity [6,7], but it has some limitations and the comprehensive effects of two forms of seismic damage cannot be taken into account. Therefore, the more reasonable two-parameter model combines the two parameters of deformation and energy. The most widely used is the two-parameter seismic damage model combined with the maximum displacement amplitude and repeated cyclic loading effect proposed by Park and Ang [8], in which the maximum displacement amplitude and repeated cyclic loading effect. Subsequently, scholars have continuously modified the model from the aspects of accuracy [9,10,11], uncertainty quantification [12,13], and different structural components [14,15,16]. Furthermore, the effects of fatigue characteristics [17,18], inelastic displacement ratio [19], restoring force [20], and three-dimensional damage [21] are further considered in the models, and these damage models are widely used in seismic damage analysis and evaluation.

A precast concrete structure is a concrete structure assembled by precast concrete members through a reliable connection such as post-cast concrete, sleeve connection, bolt connection, etc. Compared with the traditional cast-in-place concrete structure, it mainly has the advantages of high production efficiency, short construction period, good product quality, small environmental impact, sustainable development, and less labor consumption [22]. The monolithic precast frame structure is currently one of the most widely used precast concrete structural systems in China, which refers to the concrete structure that connects the precast concrete components in a reliable way and is formed as a whole with on-site post-cast concrete and cement-based grouting materials [23]. Beam-column joints are very important in the concrete frame structure, which are responsible for connecting the main load-bearing components and transmitting force [24], and the joint deformation may make a significant difference in the lateral response of RC buildings if joints are not properly designed and detailed [25]. The damage and slip of the beam rebars bonded out of the joint core can cause sudden strength decay [26], and a brittle shear failure is easy to take place [27]. For the precast concrete frame structure, the connection surface at the beam-column joint is the main difference between the precast structure and the traditional cast-in-situ structure. However, due to the existence of connection seams, the seam damage will accumulate under the repeated action of an earthquake, and the damage characteristics are different from the traditional cast-in-place concrete structure [28].

Much work has been performed on the mechanical properties of precast concrete beam-column joints. Liu et al. [29] studied different connecting methods for the precast beam-column joints and compared the seismic behavior of a precast joint with a cast-in-place one, the result showed that the precast joint was capable of matching performance in terms of peaking strength, stiffness, deformation performance of the cast-in-place connections, but the failure cracks of the precast specimen always concentrated in the junction between precast elements and post-pouring concrete. Feng et al. [30] studied the effects of post-cast connections on seismic performance of precast concrete frame joints and suggested that the location of the connection should be at a plastic hinge length away from the beam end; the distance was proposed as 2 h. Zhang et al. [31] proposed a new kind of precast composite beam-column joints without concrete cast in place in the core area, which consisted of a precast column with a built-in steel skeleton, a precast beam with a built-in H-steel beam, and connecting parts. Chen et al. [32] studied a detachable precast concrete column joint with bolted flange plate; the results showed that reliable welding between rebars and flange plate played an important role in the seismic performance of the precast column. Just like the application of new materials in traditional concrete structures [33], some new materials have been used in the precast frame joints as well. Ma et al. [34] studied the application of lap-spliced steel bars in UHPC for connecting the beam and column in the joint, and the results showed that the shear capacity and the ductility of the joint were increased obviously. Yang et al. [35] used self-compacting concrete in monolithic precast concrete joints and studied the rational lateral pressure of this concrete for the design of the formwork. Ghayeb et al. [36] investigated the impact of using engineered cementitious composite on the performance behavior of precast beam-column joints, and the results showed that the seismic behavior of the precast joints exhibited better performance than the conventional monolithic joint.

It can be seen that although there have been a lot of research results on the seismic performance of precast concrete joints, the relevant research results on the quantitative calculation and evaluation of seismic damage of precast concrete structures are extremely limited, while the relevant results of cast-in-situ concrete structures cannot be applied directly due to the different damage characteristics. However, quantitative calculation and evaluation of seismic damage are very important for structural safety, performance-based structural analysis, and seismic reinforcement. Therefore, it is very necessary to carry out seismic damage research on precast concrete structures. In this paper, for beam-column joints of monolithic precast frame structures that are widely used in China, the author attempts to use the damage index model to calculate the damage of such joints and carry out damage evaluation according to the damage index. The seismic damage process, load-displacement relationship, and stiffness degradation of monolithic precast concrete beam-column joints are studied by cyclic loading test, and the effect of axial compression ratio is analyzed as well. This paper carried out a meaningful exploration for the accurate calculation and quantitative evaluation of seismic damage of monolithic precast concrete frame structures.

## 2. Experiment Design

### 2.1. Materials

The test materials used in this study are mainly concrete and steel bars. According to the Chinese code GB 50010-2010: Code for design of concrete structures, the strength grade of concrete is C30, and the measured material mechanical properties parameters are shown in Table 1. The type of longitudinal steel bars is HRB400, and the type of transverse steel bars is HPB300, and the measured material mechanical properties are shown in Table 2 separately.

### 2.2. Specimen Design and Fabrication

**Figure 1:**
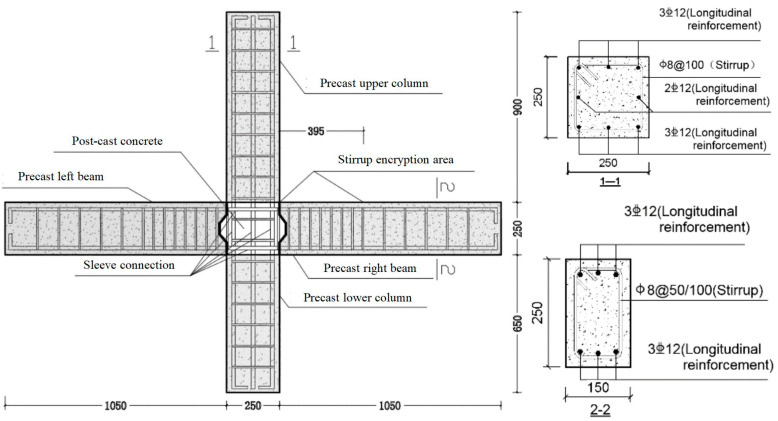
Dimensions and reinforcement of the beam-column joints/mm.

In order to accurately evaluate the damage to the beam-column joint of the precast concrete frame under the action of earthquake, the damage characteristics and mechanical properties of the beam-column joint specimen under cyclic loading were studied through quasi-static tests. Four 1:2 scale specimens of concrete beam-column joints were designed for the tests according to the Chinese codes JGJ 1-2014: Technical specification for precast concrete structures [23] and GB 50011-2010: Code for Seismic Design of Buildings [37]. Among them, ZJ0 is a cast-in-situ reinforced concrete frame joint specimen with axial compression ratio of 0.3. ZJ1, ZJ2, and ZJ3 are all monolithic precast concrete frame joints with axial compression ratios of 0.2, 0.3, and 0.4, respectively. The longitudinal reinforcements of the columns of the four specimens were 8
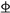
12, and the reinforcement ratio was 1.63%; the longitudinal reinforcements of the beams are all 6
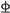
12, and the reinforcement ratio is 2.14%; the stirrup is 8 mm in diameter, and 100 mm in spacing, but in beam end encryption area is 50 mm in spacing. The size and reinforcement of the precast joint specimens are shown in Figure 1. The size and reinforcement of cast-in-place joint specimens are the same as the precast ones.

The fabrication of the precast specimens was carried out through the following steps: firstly, the precast parts, including the left and right beams and the upper and lower columns, were fabricated. In order to strengthen the shear resistance of the seam surface, shear keyways were set on the left and right beam ends, and the upper and lower column ends were chiseled. Then, the reserved steel bars of left and right beam were welded together, and the reserved steel bars of upper and lower column were connected by sleeve connectors. Finally, the post-cast concrete was poured to fill the core area. Taking into account the important influence of curing conditions on the mechanical properties and durability of concrete materials [38], the maintenance of this project was strictly carried out in accordance with the relevant specifications [23], and the curing time for these specimens was 28 days, the curing temperature and relative humidity were 20 ± 1 °C and 50 ± 5% respectively.

### 2.3. Test Device and Test Method

**Figure 2:**
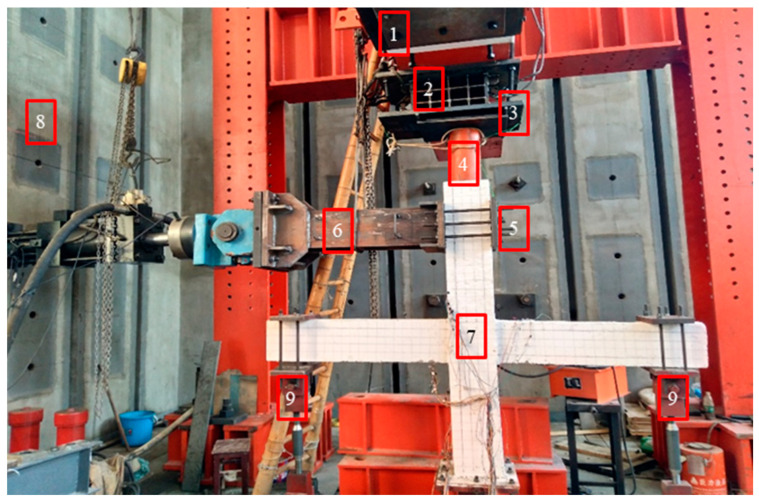
Loading device in the test: (1) Reaction beam; (2) rolling hinged support; (3) pressure sensor; (4) oil-pressure jack; (5) steel fixture for columns; (6) electrohydraulic servo actuator; (7) concrete joint; (8) back strength wall; (9) steel fixture for beams.

According to different loading points, there are usually two kinds of quasi-static loading modes for beam-column joint; one is loading at beam ends, the other is loading at column top end [39]. In this test, the column top end loading method was adopted, and the devices used for the loading are shown in Figure 2. It can be seen from Figure 2 that the column top end is clamped by a steel fixture and then hinged to the loading end of the electrohydraulic servo actuator; the other end of the actuator is fixed on the back strength wall. The column bottom end is fixed on a steel bearing with a spherical hinge, while the beam ends are fixed on the steel bearing through a steel-hinged fixture, and the steel bearing can be reliably connected with the ground groove by using anchor bolts.

**Figure 3:**
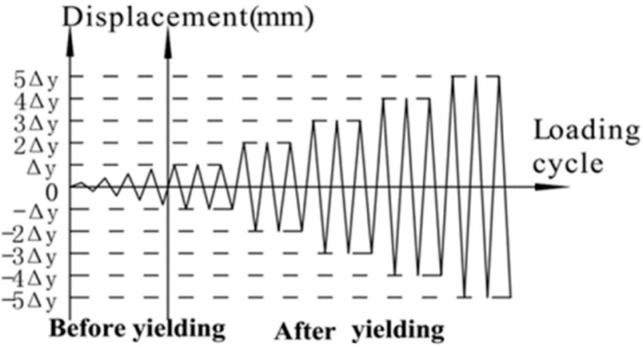
The diagram of loading process.

Displacement loading was used in this test. Before the lateral loading, the vertical load provided by the oil-pressure jack was applied on the column top and was kept to be constant. When applying lateral loads, the electrohydraulic servo actuator was used to provide the lateral cyclic loads on the column top end, and the loading was controlled by displacement. The loading increment for each cycle was set to be 3 mm before the specimen yielded, and the cycle was repeated only once. Then the yield displacement Δ_y_ was set to be the increment for each cycle and repeated 3 times after the specimen yielded. When the lateral load decreased to 85% of the peak load, it meant the test joint was destroyed, and the loading process finished. The loading process is shown in Figure 3. The horizontal load at the end of the column was automatically recorded by the actuator control system made by Hongshan, Tianshui, China and the vertical dead load at the top of the upper column was recorded by the pressure sensor provided by Hongshan, Tianshui, China as well. Displacement gauges were arranged at the top end of the upper column along the horizontal direction and at the beam ends along the vertical direction to collect the displacement of the beam and column. Interlaced displacement meters are arranged in the core area of the joint to measure the relative elongation or to shorten the diagonal of the core area to calculate the shear deformation of the core area. Traditional strain gauges are used to measure the strain of longitudinal bars and stirrups in the core area of beam-column joints, and multi-channel dynamic recorder is used to collect data. Before the formal loading starts, load to 10 kN to check whether each measuring instrument is working normally. After the formal loading starts, the horizontal displacement at the column top end is recorded by the actuator control system, the corresponding horizontal load is recorded simultaneously, the load-displacement curve is drawn, and the crack development and strain are observed as well. During the loading process, the vertical load is recorded in real time through the load sensor to ensure that the vertical load is constant.

## 3. Test Results

### 3.1. Damage Process of Specimen

**Figure 4:**
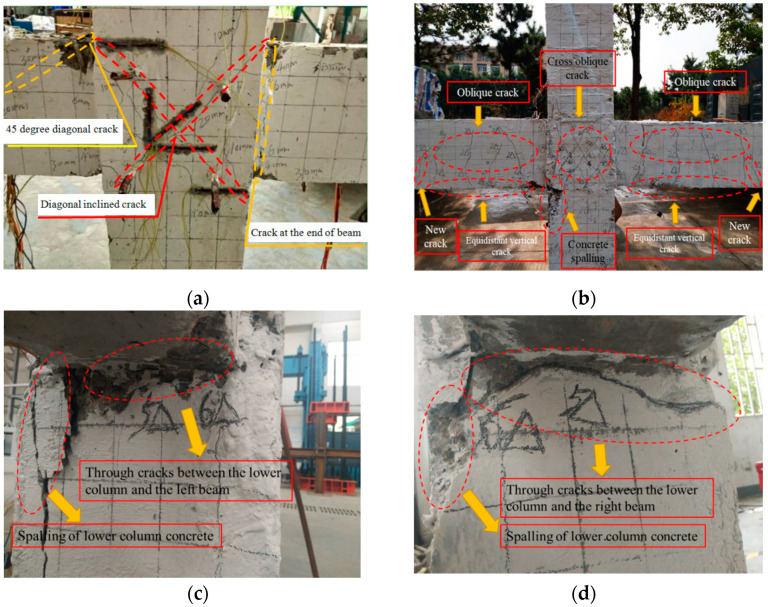
Failure modes of specimens: (**a**) Failure characteristics of ZJ0; (**b**) Failure characteristics of ZJ2; (**c**) Through cracks on the left beam and lower column of ZJ2; (**d**) Through cracks on the right beam and lower column of ZJ2.

Under low cyclic loading, the failure process and damage development mode of the precast joint specimens are basically the same. Specimen ZJ2, with an axial compression ratio of 0.3, is taken as an example and compared with the cast-in-situ specimen ZJ0 to illustrate the seismic damage evolution process of monolithic precast frame joints. According to the test, the seismic damage of monolithic precast frame joints can be roughly divided into five stages. The failure mode of the specimen is shown in Figure 4.

(1) Non-damage stage: At the initial stage of loading (the displacement is less than Δ_y_), small cracks appear in the concrete at the beam end of specimen ZJ0 and specimen ZJ2 and tend to be stable. The strain of steel bars in the core area of the beam, column, and the joint is small and in the elastic stage. At this time, the deformation of the beam and column is very small, and the strength and stiffness of the specimens have no obvious change.
(2) Initial damage stage: When the cyclic loading displacement is continued to Δ_y_, the fine bending cracks of concrete at the beam end of ZJ0 gradually develop into long oblique cracks. The growth rate of stress and strain of the steel bar in the core area of specimen ZJ2 is accelerated, and the concrete strain in the plastic hinge area of the beams increases sharply, resulting in microcracks, and several equal spacing vertical cracks appear one after another. The cracks of both joints begin at the beam end, which is consistent with the damage characteristics of a strong column and weak beam joints.
(3) Mixed damage development stage: multiple diagonal cracks appeared in the core area of specimen ZJ0 under 2Δ_y_ cyclic loading. Similarly, the stress and strain of the steel bar in the core area of specimen ZJ2 continued to increase, and the cracks in the plastic hinge area of the beams extended to the joint core area and developed from vertical cracks to oblique cracks. When the loading is continued to 3Δ_y_, the diagonal cracks of specimen ZJ0 develop to be the main cracks through the core area, and new diagonal cracks appear at the beam end. As to specimen ZJ2, new cracks began to appear at the far end of the left and right beams, old cracks continued to extend, and the width gradually increased. At the same time, the inclined cross cracks appear in the core area of the joint, and then the inclined cracks divide the concrete in the core area into several diamond blocks and extend to the lower column.
(4) Stable damage development stage: When the cyclic loading is continued to 4Δ_y_, the main crack of specimen ZJ0 widens, and the cracks in the core area and beam end increase. As to specimen ZJ2, the longitudinal reinforcement in the core area is close to yielding, the cross diagonal crack in the core area extends to the end of the lower column, and the concrete peels in a large area. Subsequently, there is a separation between the beam end, the column end, and the core area of the joint.
(5) Damage failure stage: When loading continues to the 5Δ_y_ and 6Δ_y_ cycles, the concrete at the core area and beam end of specimen ZJ0 fall off in a large area, and the bearing capacity decreases suddenly, and finally, the concrete is crushed and destroyed. As to specimen ZJ2, the longitudinal reinforcement in the core area entered the strengthening stage, and the stirrup gradually yielded. The cracks at the connection seams between the left and right beams and the upper column continue to extend, but the crack width is small, the crack width at the seams with the lower column gradually increases, and the concrete continues to peel off, and the damage continues to grow. Finally, a through-and-wide crack is formed (Figure 4c,d). At this time, the beam-column joint loses its bearing capacity.

From the above analysis, it can be seen that the failure mode of cast-in-situ specimen is the shear failure of the core area, which is occurred after the flexural yielding of the beam, and this kind of failure mode usually occurs when the column-beam flexural strength ratio is relatively high [25]. As to the precast ones, the failure mode is mainly the connection seam failure, and the weakest part is the connection seam between the beam end and the lower column. This kind of failure mode is due to the existence of the connection seam, which makes the stiffness distribution of the beam-column joint uneven, and the cracks are easy to concentrate at the connection seam [40].

### 3.2. Load-Deformation Curve

**Figure 5:**
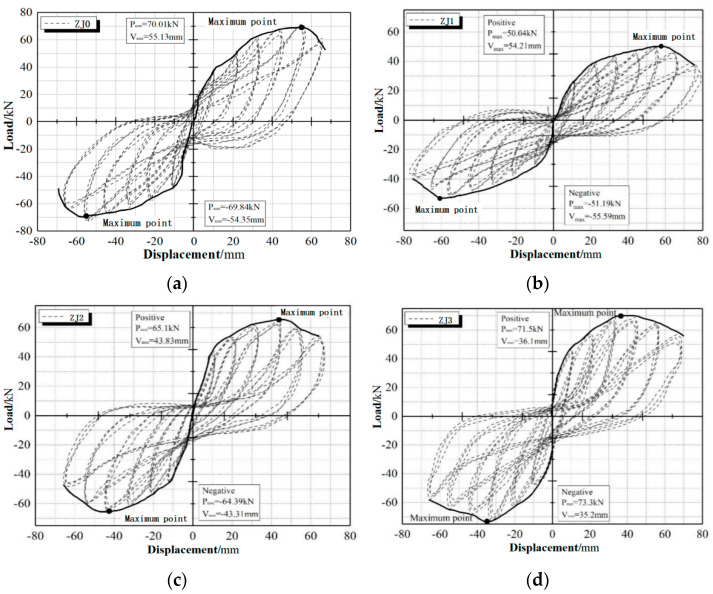
Hysteresis curves and skeleton curves of the specimens: (**a**) ZJ0; (**b**) ZJ1; (**c**) ZJ2; (**d**) ZJ3.

Figure 5 shows the hysteresis curves (dashed lines) and skeleton curves (solid lines) of the joint specimens under low cyclic loading. The hysteresis curve of the cast-in-situ specimen ZJ0 (Figure 5a) is “Z-shaped” and has an obvious pinching phenomenon, which indicates that the longitudinal reinforcement of the joint slips obviously [41]. Compared with ZJ0, the pinching phenomenon of monolithic precast joint specimen ZJ2 is improved a little (Figure 5c). In addition, comparing the skeleton curves in the above two figures, it can be seen that the displacement corresponding to the failure load of ZJ2 is smaller than that of ZJ0, and the peak load is also reduced to about 92% of the cast-in-situ specimen ZJ0.

From the above pictures, it can be concluded that the load-displacement curve of a precast monolithic joint has the following characteristics:

(1) Before the specimen yields, the initial horizontal load is small, the hysteresis loop is a spike-type, the enclosure area is small, and the skeleton curve changes linearly. At this time, the specimen damage is small. With the increase in the hysteretic curve bending, the hysteretic loop area also increases. The specimen enters the elastoplastic stage, and the concrete cracks appear in the closure and the opening phenomenon and gradually appear obvious damage. The skeleton curves show that specimen ZJ1 (axial compression ratio is 0.2), specimen ZJ2 (axial compression ratio is 0.3), and specimen ZJ3 (axial compression ratio of 0.4) yield 25.71 kN, 42.33 kN, and 46.64 kN, and the corresponding yielding displacements are 10.2 mm, 10.53 mm and 10.84 mm separately. The yielding load increases with the axial compression ratio, and the yielding displacement is similar.
(2) After the specimen yields, as the cyclic load increases continuously, both the slopes of the hysteresis curve and skeleton curve decrease. Due to the slip of the steel bar, the stiffness of the specimen degenerates, and the hysteresis curve appears to be a pinching phenomenon, and there is an obvious yield point on the skeleton curve. As concrete cracks continue to expand, the specimen damage begins to increase. The larger the axial compression ratio is, the larger the initial secant modulus and the more saturated the hysteresis loop. The skeleton curves show that the peak loads of specimen ZJ1 (axial compression ratio is 0.2), specimen ZJ2 (axial compression ratio is 0.3), and specimen ZJ3 (axial compression ratio is 0.4) reach 50.04 kN, 65.11 kN, and 71.50 kN, respectively, and the corresponding peak displacements are 54.21 mm, 43.83 mm and 36.10 mm. Compared with specimen ZJ1, the ultimate bearing capacity of specimen ZJ2 and specimen ZJ3 are increased by 30.1% and 42.9%. This is because the pressure applied to the core area of the joint can improve the strength of the concrete in the core area and can also provide a good constraint on the longitudinal reinforcement, thereby improving the bearing capacity of the joint [40]. Thus, the greater the axial compression ratio, the greater the above effect.
(3) After the specimen reaches the peak load, the hysteretic loop area increases continuously with the increase in the loading displacement, showing a good capacity for dissipating energy. Under the same cyclic load, the hysteresis loop of the previous loading is larger than that of the latter, and the maximum load in the latter loading is also reduced, indicating that the specimen has strength degradation and energy dissipation capacity reduction. The sliding section of the skeletal curve is steeper due to the reduced deformation capacity. The damage to the specimen continues to accumulate until the specimen is destroyed.

### 3.3. Stiffness Degradation Curve

Stiffness degradation refers to the characteristic that the stiffness of a structure or component decreases with the increase in cycle number under the condition of constant displacement amplitude. This is because, with the increasing times of load cycles, the damage to the structure continues to accumulate, resulting in the continuous degradation of structural stiffness. Excessive stiffness degradation can significantly reduce the mechanical properties of the structure, which can cause great structure deformation under even a small load. Loop stiffness *K_j_* is commonly used to characterize the stiffness degradation of components, and its expression is shown in Equation (1) [42].

(1) $$K_{j}=\sum_{j=1}^{n}P_{ji}/\sum_{j=1}^{n}\Delta_{ji}$$

where *P_ji_* means the maximum load applied on the column top in the *i*th (i = 1, 2, 3……) cycle under the *j*th loading step; Δ*_ji_* means the displacement corresponding to *P_ji_*.

**Figure 6:**
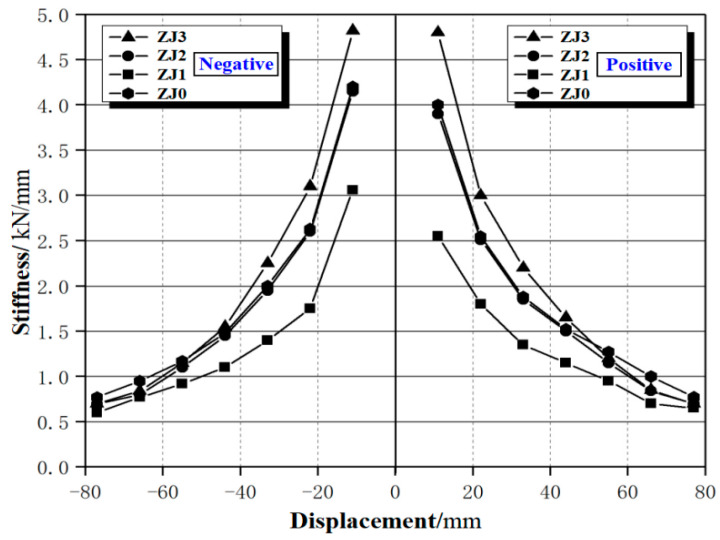
Stiffness degradation of the specimens.

Figure 6 shows the stiffness degradation of the specimens, and the stiffness values are shown in Table 3. It can be seen from Figure 6 and Table 3 that compared with the cast-in-situ specimen ZJ0, the initial stiffness of the precast specimen ZJ2 is slightly smaller, and the stiffness degradation is basically the same, but after the loading displacement reaches 4Δ_y_ (Displacement corresponding to the peak load of ZJ2), the stiffness degradation rate of ZJ2 is significantly faster than that of ZJ0, which is due to the existence of seam surfaces between the precast components, and the damage of the seam surfaces has accumulated to a certain extent, resulting in a faster decrease in the overall stiffness of the precast specimens than the cast-in-situ specimen.

Comparing the stiffness degradation of the three precast joints ZJ1, ZJ2, and ZJ3 with different axial compression ratios, we can see that:

(1) The larger the axial compression ratio, the greater the initial stiffness of the specimen. When loading displacement reaches the yield displacement, the stiffness of specimens ZJ1, ZJ2, and ZJ3 are 2.54 kN/mm, 3.90 kN/mm, and 4.83 kN/mm, respectively, and the stiffness of ZJ3 (axial compression ratio is 0.4) is about 1.9 times higher than that of ZJ1(axial compression ratio is 0.2). This is also due to the beneficial effect of axial pressure on the strength of concrete in the core area and longitudinal reinforcement constraints.
(2) After the precast specimens yield, the stiffness decreases rapidly, and the larger the axial compression ratio, the faster the stiffness degradation rate. As the loading displacement continues to increase, the stiffness degradation tends to be gentle, but the specimen with a larger axial compression ratio still has higher stiffness. For specimens with faster stiffness degradation, the load increases more slowly under the same loading displacement.
(3) When the stiffness of specimens ZJ1, ZJ2, and ZJ3 is 0.92 kN/mm, 1.49 kN/mm, and 1.98 kN/mm, respectively, the load reaches the peak value, and the corresponding stiffness of the specimens at this time decreases 36.4%, 37.1% and 46.0% of the yield stiffness, respectively. Afterward, the stiffness degradation of the three specimens tends to be gentle, and the stiffness of specimen ZJ1 decreases more slowly than that of specimens ZJ2 and ZJ3. The above phenomenon shows that the increase in the axial compression ratio will aggravate the development of damage in the core area and the connection surfaces, resulting in a more rapid decrease in the stiffness of the joint specimen.

### 3.4. Damage Analysis Based on Modified Park–Ang Model

Based on the damage test results, Kunnath [43] proposed a modified Park–Ang model, and the damage index *D* can be calculated as Equation (2):

(2) $$D=\frac{\delta_{m,i}-\delta_{y}}{\delta_{f}-\delta_{y}}+\beta \frac{E_{h}}{F_{y}\delta_{f}}$$

where *δ_m,i_* is the maximum displacement of the *i*th loading cycle, and *δ_y_* and *δ_f_* mean the yield displacement and the ultimate displacement under monotonic load. Moreover, *E_h_* is accumulated hysteretic energy under cyclic load, and *F_y_* is the yield force. The value of *β* is the hysteretic energy factor; when *β* = 0.05, the modified Park–Ang model can better reflect the damage process of beam-column joints [44].

**Figure 7:**
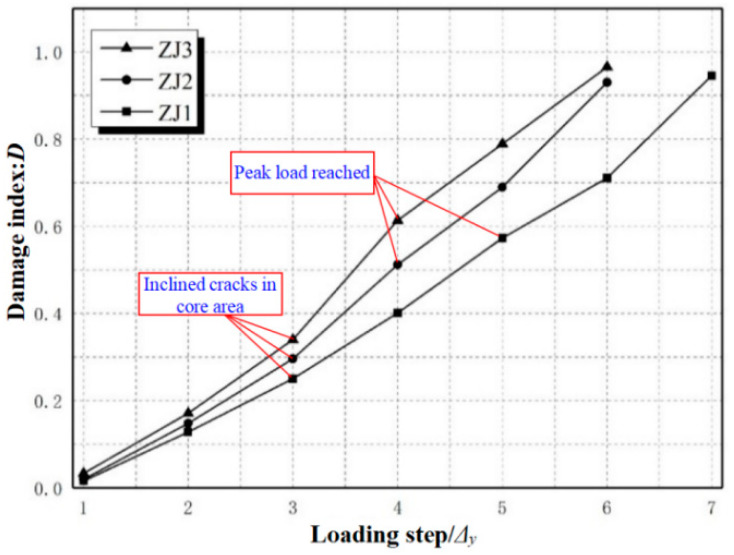
Damage evolution curves of the precast specimens.

The hysteretic energy can be taken as the hysteresis loop area of each loading step, and the lateral displacement of the top column corresponding to the applied load decreasing to 85% peak load is assumed to be the ultimate displacement for the joint specimen. Based on the test results, the loading displacement of each step 1Δ_y_, 2Δ_y_, 3Δ_y_ … are taken as the abscissa, and the corresponding damage index D1, D2, D3, … are taken as the ordinate. Through the stiffness degradation analysis, it can be seen that the damage development of the cast-in-situ specimen KJ0 and the precast specimen KJ2 are basically the same. Therefore, only the damage of the three precast joint specimens is calculated and analyzed here. Based on Equation (2), the calculated damage index values of the precast specimens are shown in Table 4, and the damage evolution curves are shown in Figure 7.

It can be seen from Table 4 and Figure 7 that the damage indexes of the three specimens increase with the increase in loading displacement. When the loading displacement reaches Δ_y_, the damage indexes of ZJ1, ZJ2, and ZJ3 are very small, which are only 0.016, 0.021, and 0.033, respectively. At this time, the corresponding experimental phenomenon is that there are small cracks at the beam end, and the reinforcement strain is small. The specimen is in the elastic stage and is basically intact.

When the loading displacement reaches 2Δ_y_, the damage indexes of ZJ1, ZJ2, and ZJ3 are 0.128, 0.148, and 0.171, respectively. At this time, the corresponding experimental phenomenon is that small cracks appear in the plastic hinge zone of the beam, and the small cracks at the end of the beam develop into long oblique cracks. The stiffness degradation of the specimen is obvious, and the specimen is in a slight damage stage.

When the loading displacement reaches 3Δy, the damage index of ZJ3 reaches 0.375, which is 1.27 times of ZJ2 and 1.5 times of ZJ1, indicating that the axial pressure has a significant effect on the damage development. At this time, the corresponding experimental phenomenon is that there are many vertical and oblique cracks in the core area of the joint, and the cracks between the beam-column connection interface continue to expand, and the specimen is in the middle damage stage.

With the continued increase in loading displacement, the slope of the damage evolution curve becomes larger, and the damage index begins to accelerate growth, especially for specimen ZJ3, with the largest axial compression ratio. The damage index of ZJ3 at 4Δy reaches 1.64 times the index at 3Δy. In addition, ZJ3 and ZJ2 reached the peak load before loading to 4Δy, and the ZJ1 specimen reached its peak load shortly after loading to 5Δy. In the loading displacement stage, from 3Δy to 5Δy, the maximum damage index of the specimen reaches 0.79 (ZJ3). The corresponding experimental phenomenon is that the main cracks of the specimen become wider, the longitudinal reinforcement in the core area is close to yielding, and the concrete is spalling in a large area. The contact between the beam end, column end, and the core area of the joint is separated. The stiffness of the specimen is reduced to less than half of the stiffness when the yield displacement is reached and the specimen is in the severe damage stage.

As the loading displacement continues to increase, the damage indexes of the three specimens are all above 0.9. The concrete in the core area is crushed, and the stirrup yields, forming a wide diagonal crack. The beam-column joint loses its bearing capacity, and it is in a complete failure stage.

The above studies show that the damage index model adopted in this paper can be used for the damage calculation of the monolithic precast joints and can accurately reflect the damage characteristics.

### 3.5. Damage States

In order to distinguish different degrees of seismic damage, damage states are often used according to the damage index calculation and the damage characteristics. On the basis of the test results and the index calculation results, the damage process of the precast beam-column joints can be divided into five stages: little or no damage, a few damages, damages developing quickly, damages developing stably, serious damages, and the joint is destroyed. Therefore, the corresponding damage states are basically intact, slight damage, moderate damage, severe damage, and complete failure. Referring to the existing literature [45] and based on the test results and damage indexes, this paper presents a quantitative evaluation table for damage states of the beam-column joints in the monolithic precast concrete frame structure, as shown in Table 5.

It can be seen from Table 5 that the damage index model adopted in this paper can quantitatively calculate the damage state of the monolithic precast concrete joints, and through the conclusion of the previous damage test and stiffness degradation analysis, the corresponding relationship between damage states and damage characteristics is established, so as to realize the quantitative damage evaluation of this kind of joints. In this paper, five damage states are defined, which are similar to the damage states defined in Reference [45]. However, due to the damage to the connection seams in precast beam-column joints, the damage index value and damage characteristics are different from those in Reference [45].

## 4. Conclusions

In this paper, the seismic damage evaluation of beam-column joints in monolithic precast concrete frame structures is studied by cyclic loading test and damage analysis. The main conclusions are as follows:

1. The damage process of precast joints can be divided into five stages, and the connection seam in precast joints can significantly affect damage development. Compared with the shear failure of the core area in the cast-in-situ specimen, the failure mode of the precast joint is mainly the connection seam failure. While for the precast joints, the axial compression ratio has no obvious effect on the failure modes.
2. Compared with the cast-in-situ joint, the pinching phenomenon of the monolithic precast joint is improved a little, but the ultimate displacement is smaller, and the peak load is only 92% of the cast-in-situ one. As for precast joints, a certain amount of axial pressure can effectively improve the bearing capacity. Both the yielding load and peak load of the precast joints increase obviously with the increase in axial compression ratio. Compared with specimen ZJ1, the peak load of specimens ZJ2 and ZJ3 increased by 30.1% and 42.9%, respectively.
3. The stiffness degradation of precast joints is basically consistent with that of cast-in-situ joints, but when the loading reaches a certain degree, the stiffness degradation of precast components will be faster due to the accumulation of damage at the connection seams. As for the precast joints, a larger axial compression ratio can bring higher initial stiffness but faster stiffness degradation. When reaching peak load, the stiffness of ZJ3 with the highest axial compression ratio decreases 46.0% compared with the yielding stiffness, while ZJ1 only decreases 36.4%.
4. The damage index model adopted in this paper can accurately reflect the damage characteristics of the monolithic precast joints. The damage indexes are all smaller than 0.1 before the joints yield, while the indexes are beyond 0.8 when the joints enter the failure stage. The damage states of the precast beam-column joints can be defined as five levels according to a different damage index value. The presented quantitative evaluation table established the corresponding relationship between damage states and damage characteristics, thus realizing the quantitative damage evaluation of this kind of joint.

## Figures and Tables

**Table 1:** Mechanical properties of concrete.

Concrete Grade	*f*_cu_/MPa	*f*_c_/MPa	*f*_t_/MPa	*E*_c_/MPa
C30	38.6	25.4	2.31	30,218

*f*_cu_ stands for the cube compressive strength, *f*_c_ stands for the axial compressive strength, *f*_t_ stands for the axial tensile strength, *E*_c_ stands for the elastic modulus.

**Table 2:** Mechanical properties of steel bars.

Grade	*f*_y_/MPa	*f*_u_/MPa	*E*_s_/GPa	*δ*_gt_/%	ε_y_/10^−^^6^
HRB400	396	510	201	23.2	2113
HPB300	342	425	200	22.8	2080

*f*_y_ stands for the yield strength, *f*_u_ stands for the ultimate strength, *E*_s_ stands for the elastic modulus, *δ*_gt_ stands for the elongation, ε_y_ stands for the yield strain.

**Table 3:** Stiffness of the specimens in the loading process (Unit: kN/mm).

Specimen	Loading Direction	Loading Displacement						
		1Δy	2Δy	3Δy	4Δy	5Δy	6Δy	7Δy
ZJ0	Positive	4.02	2.54	1.88	1.49	1.16	1.02	0.72
	Negtive	4.21	2.65	1.99	1.51	1.19	0.96	0.72
ZJ1	Positive	2.54	1.82	1.35	1.12	0.92	0.72	0.53
	Negtive	3.07	1.77	1.42	1.08	0.92	0.72	0.53
ZJ2	Positive	3.9	2.52	1.88	1.49	1.11	0.85	0.63
	Negtive	4.18	2.64	1.96	1.48	1.09	0.83	0.62
ZJ3	Positive	4.83	2.97	2.21	1.58	1.16	0.85	0.68
	Negtive	4.85	3.08	2.26	1.52	1.17	0.90	0.67

**Table 4:** Damage index values of the precast specimens.

Specimen	1Δ_y_	2Δ_y_	3Δ_y_	4Δ_y_	5Δ_y_	6Δ_y_	7Δ_y_
ZJ1	0.016	0.128	0.25	0.401	0.573	0.711	0.925
ZJ2	0.021	0.148	0.296	0.498	0.75	0.92	/
ZJ3	0.033	0.171	0.375	0.614	0.79	0.93	/

**Table 5:** Damage states of the monolithic precast beam-column joints.

Damage States	Damage Index	Structure State	Damage Feature
Basically intact	0~0.1	Serviceable	/
Slight damage	0.1~0.2	Repairable	small cracks in the plastic hinge zone of the beam; long oblique cracks at beam end; obvious stiffness degradation.
Moderate damage	0.2~0.4	Repairable	vertical and oblique cracks in the core area of the joint; cracks expanding in the connection interface; stiffness is less than the yielding stiffness.
Severe damage	0.4~0.8	Irreparable	wide main cracks; longitudinal reinforcement in the core area yields; concrete is spalling off in a large area; connection between beam end, column end and core area is separated; smaller structure stiffness and slower stiffness degradation.
Complete failure	0.8~1.0	Total loss	concrete in the core area is crushed; stirrup yields, throughout diagonal crack formed.

## Data Availability

Not applicable.
